# Exercise restores brain insulin sensitivity in sedentary adults who are overweight and obese

**DOI:** 10.1172/jci.insight.161498

**Published:** 2022-09-22

**Authors:** Stephanie Kullmann, Thomas Goj, Ralf Veit, Louise Fritsche, Lore Wagner, Patrick Schneeweiss, Miriam Hoene, Christoph Hoffmann, Jürgen Machann, Andreas Niess, Hubert Preissl, Andreas L. Birkenfeld, Andreas Peter, Hans-Ulrich Häring, Andreas Fritsche, Anja Moller, Cora Weigert, Martin Heni

**Affiliations:** 1 Institute for Diabetes Research and Metabolic Diseases of the Helmholtz Center Munich at the University of Tübingen, Tübingen, Germany.; 2 German Center for Diabetes Research (DZD e.V.), Neuherberg, Germany.; 3 Department of Internal Medicine, Division of Endocrinology, Diabetology and Nephrology, Eberhard Karls University Tübingen, Tübingen, Germany.; 4 Institute for Clinical Chemistry and Pathobiochemistry and; 5 Department of Sports Medicine, University Hospital Tübingen, Germany.; 6 Interfaculty Research Institute for Sport and Physical Activity, University of Tübingen, Tübingen, Germany.; 7 Department of Radiology, Section on Experimental Radiology, University Hospital Tübingen, Germany.; 8 Institute for Diabetes and Obesity, Helmholtz Diabetes Center, Helmholtz Zentrum München, German Research Center for Environmental Health (GmbH), Neuherberg, Germany.; 9 Division of Endocrinology and Diabetology, Department of Internal Medicine I, Ulm University Hospital, Ulm, Germany.

**Keywords:** Metabolism, Neuroscience, Adipose tissue, Insulin signaling, Neuroimaging

## Abstract

**BACKGROUND:**

Insulin resistance of the brain can unfavorably affect long-term weight maintenance and body fat distribution. Little is known if and how brain insulin sensitivity can be restored in humans. We aimed to evaluate the effects of an exercise intervention on insulin sensitivity of the brain and how this relates to exercise-induced changes in whole-body metabolism and behavior.

**METHODS:**

In this clinical trial, sedentary participants who were overweight and obese underwent an 8-week supervised aerobic training intervention. Brain insulin sensitivity was assessed in 21 participants (14 women, 7 men; age range 21–59 years; BMI range 27.5–45.5 kg/m^2^) using functional MRI, combined with intranasal administration of insulin, before and after the intervention.

**RESULTS:**

The exercise program resulted in enhanced brain insulin action to the level of a person of healthy weight, demonstrated by increased insulin-induced striatal activity and strengthened hippocampal functional connectivity. Improved brain insulin action correlated with increased mitochondrial respiration in skeletal muscle, reductions in visceral fat and hunger, as well as improved cognition. Mediation analyses suggest that improved brain insulin responsiveness helps mediate the peripheral exercise effects leading to healthier body fat distribution and reduced perception of hunger.

**CONCLUSION:**

Our study demonstrates that an 8-week exercise intervention in sedentary individuals can restore insulin action in the brain. Hence, the ameliorating benefits of exercise toward brain insulin resistance may provide an objective therapeutic target in humans in the challenge to reduce diabetes risk factors.

**TRIAL REGISTRATION:**

ClinicalTrials.gov (NCT03151590).

**FUNDING:**

BMBF/DZD 01GI0925.

## Graphical abstract

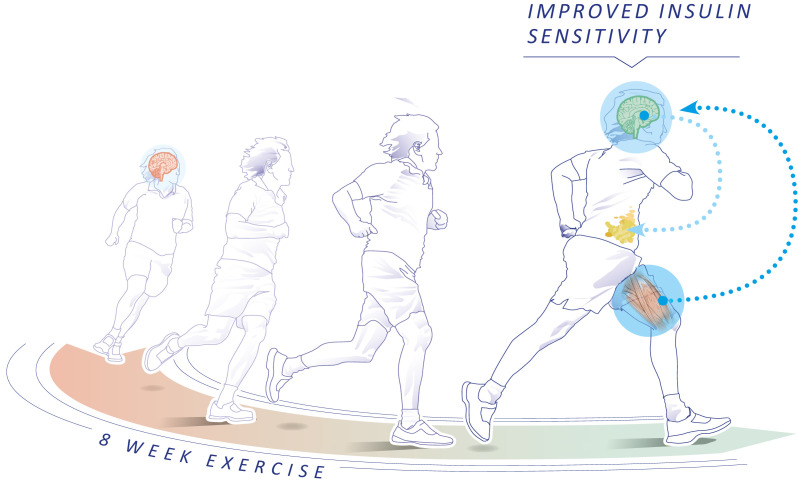

## Introduction

Insulin resistance (IR) is the hallmark feature of obesity and type 2 diabetes (T2D) with detrimental effects in the periphery (1), as well as the CNS (2). Insulin acts on neural circuits to control systemic metabolism (3) and body weight (4) by activating its receptors expressed in neurons (5) and nonneuronal cells (6, 7). In the insulin resistant state, in rodents, altered insulin-evoked activity is present in the hypothalamus, cortex, hippocampus, amygdala, cerebellum, striatum and midbrain (2, 8). Disruption of insulin receptors in the brain results in obesity-associated IR (4, 7), whereas restoration of brain insulin receptor function prevents diabetes in the preclinical animal model (9). Human brain imaging studies show that central insulin action affects region-specific activity and functional connectivity (FC) of the human brain with subsequent effects on cognition, eating behavior and metabolism (for review see refs. 2, 10). These diverse central insulin responses largely depend on its action in the hypothalamus, amygdala, hippocampus, striatum and parts of the insula and prefrontal cortex (PFC) (11–24). Specifically, central insulin action curbs food intake, improves mood and memory function (for review, see ref. 10), and modulates whole-body insulin sensitivity (25, 26), endogenous glucose production (16, 27), pancreatic insulin secretion (28) and lipolysis from visceral adipose tissue (VAT) (29). All effects in concert govern postprandial metabolism of the entire organism.

In people with brain IR, central insulin action can no longer properly regulate peripheral energy metabolism and behavior (2, 30, 31). These individuals have an unfavorable fat distribution with elevated VAT and free fatty acids (13, 32–34). Moreover, brain IR results in a greater regain of fat mass after a lifestyle intervention (32) and is linked to a considerable risk for cognitive decline (30, 35, 36). Consequently, overcoming brain IR may have a key role in aiding to prevent visceral obesity, T2D, and related neurometabolic diseases.

However, it remains unclear whether it is possible to reverse brain IR in humans. In the periphery, compromised insulin sensitivity can be restored by exercise by increasing skeletal muscle glucose uptake and thereby improving glycemic control (37, 38). Moreover, regular physical activity enhances long-term weight maintenance (39–41) and can markedly reduce the risk for T2D (42, 43). Underlying mechanisms include improved whole-body fat oxidation and decreasing visceral fat (44). As a key determinant of long-term brain health (45), regular physical activity has the potential to improve brain function, irrespective of health status and age (46).

Animal models show the ability of exercise to promote insulin signaling in the brain with subsequent reduction in body weight (47). However, no study thus far, to our knowledge, has investigated how exercise affects brain insulin responsiveness in humans. This is of great importance due to the frequent prevalence of brain IR in overweight and obese individuals (32).

Here, we investigated whether an 8-week supervised aerobic exercise training intervention changes brain insulin action, assessed through functional MRI (fMRI), in sedentary adults who are overweight and obese and how this relates to exercise-induced changes in whole-body metabolism, perceived hunger, and cognitive performance. In response to the exercise intervention, we specifically hypothesized that the improvement in insulin action would be evident as a heightened neural responsivity to intranasal insulin. This was assessed via cerebral blood flow (CBF) and resting-state FC measurements in previously identified insulin-responsive brain regions (for review, see refs. 2, 8).

## Results

### Exercise effect on fitness and peripheral metabolism.

**Figure 1:**
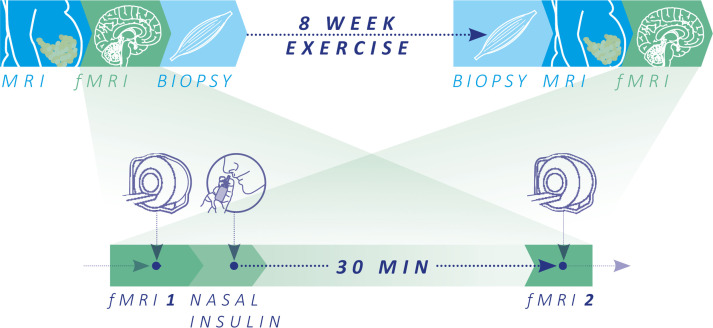
Schematic overview of the study and major test procedures. Whole-body MRI and functional MRI (fMRI) of the brain and skeletal muscle biopsies were acquired on 3 separate days before and after the 8-week exercise intervention. fMRI was assessed following overnight fasting (10 hours minimum) to investigate brain insulin action using cerebral blood flow (CBF) and functional connectivity (FC) in the fasted state (fMRI-1) and 30 minutes after nasal insulin spray application (fMRI-2). To quantify brain insulin action, ΔCBF and ΔFC were calculated (fMRI-2 – fMRI-1).

The participants completed an 8-week supervised aerobic endurance exercise intervention program at 80% peak oxygen uptake (VO_2_peak) (Figure 1). After the intervention, participants improved in cardiorespiratory fitness and mitochondrial respiratory capacity in skeletal muscle fibers and showed reduced total adipose tissue and fasting plasma glucose levels (Table 1 and Supplemental Table 1; supplemental material available online with this article; https://doi.org/10.1172/jci.insight.161498DS1). As expected from our previous results (48, 49), a large individual variance in the response in peripheral insulin sensitivity was found (assessed as HOMA-IR in the fasting state and as Matsuda index from the oral glucose tolerance test [OGTT]), resulting in no significant improvement over all participants.

### Exercise effect on brain insulin responsiveness assessed by CBF.

After the exercise intervention, we observed an increase in regional CBF in the midbrain and cerebellum independent of nasal insulin administration (family wise error *P* value [*P*_FWE_] < 0.05) (see Supplemental Figure 2 and Supplemental Table 2). No change in global CBF was observed (*P* = 0.193).

**Figure 2:**
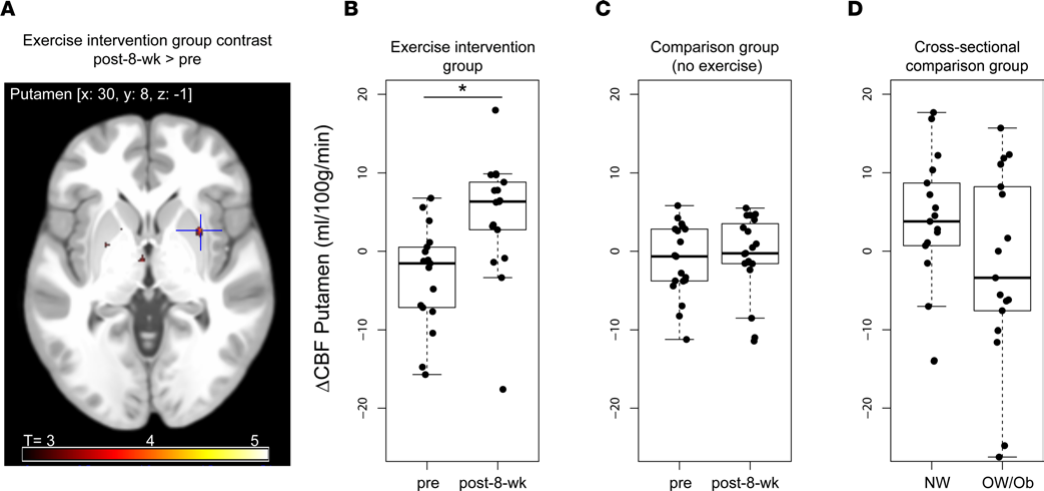
Increased brain insulin action after an 8-week exercise intervention in the putamen. (**A**) Image shows cluster in the putamen with an increase in cerebral blood flow from before to after the exercise intervention. Color map corresponds to T values (*P* < 0.001 uncorrected for display). (**B**–**D**) Box plots show change in absolute cerebral blood flow in the right putamen from before to after insulin nasal spray (ΔCBF= fMRI-2 – fMRI-1). (**B**) Before and after 8-week exercise intervention in overweight and obese individuals (*n* = 18; *P*_FWE_ < 0.05). **C** and **D** are based on previously published data sets serving as comparison groups. Before and after 8 weeks without exercise intervention, after oral placebo intake (*n* = 19) (50) (**C**), and cross-sectionally in healthy weight (*n* = 17) and overweight/obese (*n* = 17) individuals at a single time point (13) (**D**). In the plot, the box indicates the first and third quartile (25th and 75th percentile), the line in the box marks the median, and whiskers above and below indicate 1.5 × interquartile range. CBF, cerebral blood flow. *P_FWE_ < 0.05 SVC.

We next analyzed exercise effects on brain insulin responsiveness on a whole-brain level (Supplemental Figure 1). After the exercise intervention, insulin nasal spray induced a significant increase in regional blood flow in parts of the striatum (i.e., right putamen), a response not present prior to the intervention (paired *t* test of ΔCBF_pre_ versus ΔCBF_post-8-week_; *P* < 0.05, FWE correction after small volume correction [SVC]; MNI coordinates: *x*, 30; *y*, 8; *z*, –1; T = 4.05) (Figure 2, A and B, and Supplemental Table 2). No other insulin-sensitive brain regions — namely the bilateral hypothalamus, hippocampus, amygdala, insula, or PFC — showed a significant change in the response to intranasal insulin after exercise (*P* > 0.001 uncorrected, *P*_FWE_ > 0.05 SVC).

To account for the lack of a control group in the current study, we extracted the insulin response of the right putamen from the comparison group of a previously published study with the same acquisition method in response to intranasal insulin but without any exercise intervention (50). Nineteen BMI-matched individuals (9 women, 10 men; mean age 62.5 ± 8 years, mean BMI 30.9 ± 3.2 kg/m^2^) underwent CBF measurements in response to intranasal insulin at the beginning and after 8 weeks of oral placebo intake (50). In this group, insulin response in the right putamen did not change from before to after 8 weeks of oral placebo intake (*P* > 0.05; Figure 2C and Supplemental Table 3 and Supplemental Results).

Additionally, we extracted the insulin response of the right putamen in 34 age-matched participants from a cross-sectional study (13), specifically in 17 participants of healthy weight (7 women, 10 men; mean age 26.8 ± 2.8 years; mean BMI 22.8 ± 1.4 kg/m^2^) and 17 participants of overweight and obesity (8 women, 9 men; mean age 26.4 ± 2.3 years; mean BMI 30.4 ± 3.9 kg/m^2^). Prior to the exercise intervention (at baseline), participants of the current study showed a comparable central insulin response in the right putamen as the participants with overweight and obesity. After the exercise intervention, participants of the current study showed a similar increase to central insulin as persons of healthy weight (Figure 2D and Supplemental Table 3). Hence, 8 weeks of endurance exercise led to a change in the insulin response in putamen in individuals who are overweight or obese, and this is comparable with the insulin response in this brain region of healthy-weight persons (see Supplemental Results for between-group statistics).

### Exercise effect on brain insulin responsiveness assessed by FC.

We next evaluated exercise-induced changes in FC. For this purpose, we evaluated the default-mode network (DMN), as recently described (15). The DMN includes insulin-responsive regions in the PFC and temporal cortex (including the hippocampus).

**Figure 3:**
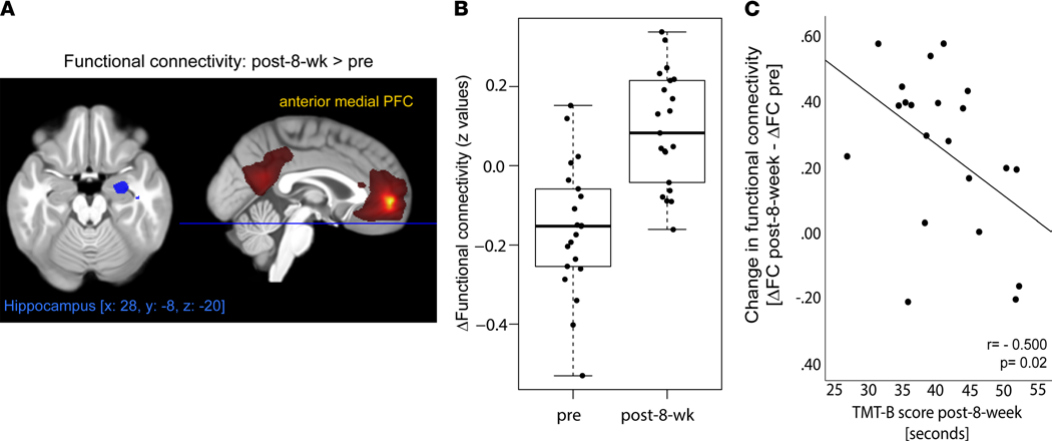
Improved brain insulin action after an 8-week exercise intervention in the right hippocampus. (**A**) Image shows cluster (in blue) in the right hippocampus with an increase in functional connectivity to the anterior medial prefrontal cortex (region in yellow) of the default mode network from before to after the exercise intervention (*P*_FWE_ < 0.05 SVC). Color map in red to yellow corresponds to group averaged default mode network at fMRI-1 (*t* test, *P*_FWE_ < 0.05). (**B**) Box plot shows change in functional connectivity between the right hippocampus and medial prefrontal cortex from before to after insulin nasal spray (fMRI-2 – fMRI-1) before and after 8-week exercise intervention (*n* = 21; *P*_FWE_ < 0.05). (**C**) Change in brain insulin action after an 8-week exercise intervention associates with cognitive function. The *y* axis displays the change in insulin action from before to after the exercise intervention (ΔFC_post-8-week_– ΔFC_pre_), and the *x* axis shows the TMT B score in seconds.

The exercise intervention per se did not significantly affect DMN FC (*P*_FWE_ > 0.05). However, in response to intranasal insulin, participants showed a significant increase in FC after the 8-week exercise intervention compared with before exercise. Specifically, insulin was able to increase FC in the DMN between the anterior medial PFC and the right hippocampus in a significantly stronger fashion after the intervention (Figure 3) (paired *t* test of ΔFC_pre_ versus ΔFC_post-8-week_; right hippocampus, T = 4.91; *P*_FWE_ < 0.05 after SVC; MNI coordinates: *x*, 28; *y*, –8; *z*, –20; left hippocampus, T = 3.6; *P* = 0.089, FWE correction after SVC; MNI coordinates: *x*, –24; *y*, –1; *z*, –1).

### Correlations between brain insulin responsiveness, cognitive function, and hunger ratings.

Participants improved in their cognitive performance from before to after exercise in the trail making test B (TMT B) (T[df = 20]= 4,8; *P* = 0.0001) but not TMT A score (T[df = 20]= 1.7; *P* = 0.09) (Supplemental Table 4).

We furthermore observed a significant correlation between the exercise-induced enhanced insulin response in FC (ΔFC_post-8-week_ – ΔFC_pre_) and cognitive function at follow-up based on the TMT B score (*r* = –0.500, *P* = 0.02, adjusted for age and BMI; Figure 3C). Hence, individuals with a more pronounced insulin responsiveness in the brain showed better cognitive flexibility after the exercise intervention, indicated by reduced time to successfully complete the TMT B task. No such correlation was observed with the TMT A score (*P* > 0.05).

**Figure 4:**
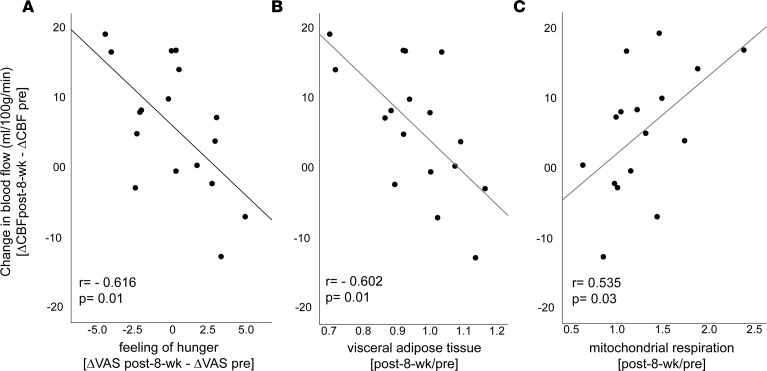
Change in brain insulin action after an 8-week exercise intervention associates with hunger ratings and metabolic measures. (**A**) The *y* axis displays the change in right putamen blood flow in response to intranasal insulin from before to after the exercise intervention (ΔCBF_post-8-week_ – ΔCBF_pre_). The *x* axis shows the change in hunger ratings in response to intranasal insulin (ΔVAS_post-8-week_ – ΔVAS_pre_). (**B**) The fold change of visceral adipose tissue from before to after the 8-week exercise intervention. (**C**) The fold change of maximal coupled skeletal muscle mitochondrial respiration in skeletal muscle fibers from before to after the 8-week exercise intervention. CBF, cerebral blood flow; VAS, visual analogue scale.

Overall, no significant exercise-induced effect was observed in perceived feeling of hunger before or after intranasal insulin application (*P* > 0.05; Supplemental Table 4). However, the exercise-induced insulin response in the putamen (ΔCBF_post-8-week_ – ΔCBF_pre_) significantly correlated with the change in perceived feeling of hunger in response to intranasal insulin (fMRI-2 – fMRI-1) (*r* = –0.616, *P* = 0.01, adjusted for age and BMI**;** Figure 4A). People with an improved insulin response in the right putamen felt less hungry after the intervention in response to intranasal insulin.

### Correlations between brain insulin responsiveness and metabolism.

Next, we studied potential associations of the exercise-induced insulin response in the putamen (ΔCBF_post-8-week_ – ΔCBF_pre_) and exercise effects on peripheral metabolism. The change in the insulin response in the right putamen significantly correlated negatively with the change in VAT (*r* = –0.602, *P* = 0.01; Figure 4B). Furthermore, the change in the insulin response in the right putamen correlated positively with the increase in skeletal muscle respiratory capacity — specifically, with the change in maximal coupled mitochondrial respiration of the skeletal muscle (*r* = 0.535, *P* = 0.03; Figure 4C), with the change in maximal uncoupled respiration (*r* = 0.588, *P* = 0.01), and with the change in fatty acid– and pyruvate-driven respiration (*r* = 0.502, *P* = 0.04). Hence, improved brain insulin responsiveness was linked to decreased visceral fat and enhanced mitochondrial respiration in skeletal muscle after the intervention. No such associations were identified with peripheral insulin sensitivity or with the amount of total adipose tissue or s.c. adipose tissue (*P* > 0.05).

### Brain insulin action as a potential mediator of exercise-induced benefits in the periphery.

Based on the correlations between the exercise-induced change in right putamen insulin action (ΔCBF_post-8-week_ – ΔCBF_pre_), metabolism, and perceived hunger, we tested, by mediation analyses, the process that underlies the observed relationships. Analyzed measures of peripheral metabolism included the fold change (after the 8-week exercise intervention/before the intervention [post-8-week/pre]) of maximal coupled skeletal muscle mitochondrial respiration and the fold change (post-8-week/pre) of the amount of VAT. The change in perceived feeling of hunger in response to intranasal insulin (change in visual analogue scale after the 8-week exercise intervention ∆VAS_post-8-week_ – ∆VAS_pre_) served as a measure for central insulin induced effect on eating behavior.

**Figure 5:**
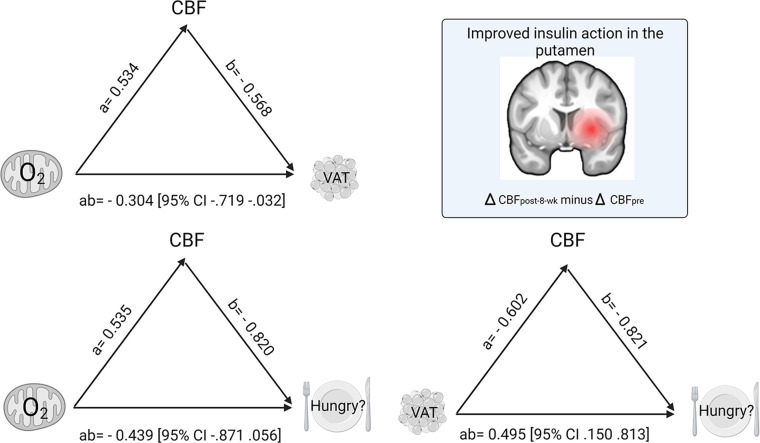
Model of exercise-promoted central insulin action as a mediator between changes in peripheral metabolism and central insulin modulated feeling of hunger from before to after an 8-week exercise intervention. Path coefficients and CIs are shown next to arrows. All variables relate to changes from before to after the 8-week exercise intervention. Brain template at the top right of the graph shows region in the striatum (i.e., right putamen), revealing a significant exercise-induced increase in central insulin action (ΔCBF_post-8-week_ – ΔCBF _pre_). In the model on the top left, path *ab* indicates the indirect effect of the change in maximal coupled mitochondrial respiration in skeletal muscle fibers on the change in VAT via the exercise-induced change in putamen insulin action. In the model on the bottom left, path *ab* indicates the indirect effect of the change in maximal coupled mitochondrial respiration in skeletal muscle fibers on the change in hunger (ΔVAS_post-8-week_ – ΔVAS_pre_) via the exercise-induced change in right putamen insulin action. In the model on the bottom right, path *ab* indicates the indirect effect of the change in VAT on hunger via the exercise-induced change in putamen insulin action. CBF, cerebral blood flow; O_2_, oxygen flux for mitochondrial respiration; VAS, visual analogue scale for hunger ratings; VAT, visceral adipose tissue.

Mediation models using the change of hunger ratings, VAT, or skeletal muscle mitochondrial respiration as the mediators did not indicate significant indirect effects. However, the analyses revealed significant indirect effects via increased putamen insulin action as a mediator (95% CI, 10,000 bootstrap samples). Specifically, the following indirect effects via increased insulin action in the putamen were observed: (a) of skeletal muscle mitochondrial respiration on VAT (*ab*= –0.304, 95% CI [–0.719 to –0.032]), (b) of mitochondrial respiration (*ab*= –0.439, 95% CI [–0.871 to –0.05]), and (c) of VAT (*ab*= 0.495, 95% CI [0.150 to 0.813]) on intranasal insulin-modulated hunger ratings. This suggests that exercise promotes metabolic and eating behavior processes via central insulin action. For more details, see schematic overview of results (Figure 5 and Supplemental Table 5).

No direct effects of change in mitochondrial respiration on changes in VAT or hunger ratings, or of change in VAT on change in hunger ratings, were observed (Supplemental Table 5).

## Discussion

Animal models and human studies show that the mesolimbic circuitry is finely tuned in response to insulin (2, 8). In people with obesity, these mechanisms are impaired, which might increase their risk of developing T2D and associated diseases (2). Our study demonstrated that an 8-week aerobic exercise training may overcome these impairments in people with obesity. We reported beneficial effects of exercise on 2 important measures of brain insulin responsiveness in the mesolimbic system. After the exercise intervention, central insulin administration was able to increase regional activity in crucial parts of the striatum and strengthen functional connections of the hippocampus. This enhanced brain insulin action was linked to improved cognitive, metabolic, and behavioral functions. Hence, our study provides the first evidence to our knowledge in humans that it is possible to influence obesity-associated brain IR in regions of the mesolimbic system with an exercise intervention.

Before the exercise intervention, the striatum was found to be unresponsive to central insulin in our participants who are overweight or obese. Their insulin-induced response in striatal blood flow was similar to what has previously been described for men who are overweight (18). After the exercise intervention central insulin responsiveness in the striatal blood flow was normalized to a level comparable with healthy lean individuals (11), with related beneficial effects on peripheral metabolism and behavior. No such improvement was seen in an overweight and obese comparison group (50) of individuals who did not participate in an exercise intervention, where reduced central insulin response in the right putamen did not change over an 8-week time period.

The improved striatal insulin sensitivity after the intervention was closely linked to the exercise-induced altered visceral fat mass and hunger ratings. Our results suggest that exercise can restore the functional response in specific brain regions that aid in insulin’s ability to regulate appetite, even in persons with obesity, in whom this control system is normally impaired. Concurrently, brain insulin action was recently detected as an important determinant for the long-term course of body weight and body fat distribution (32). Besides hypothalamic control (4), accumulating evidence suggests that brain-derived modulation of whole-body metabolism depends on intact dopamine signaling in the striatum (8, 10, 16, 25, 51, 52). This hypothesis is supported by the observation that dopamine levels in the striatum are linked to peripheral glucose metabolism (53) and that intranasal insulin administration directly modulates striatal dopamine levels (54). In line with this conceptualization, research in animals revealed a complex regulation of dopaminergic transmission by insulin (47), and exercise increased dopamine release in the striatum (55). In rats, exercise boosted central insulin’s ability to regulate dopamine levels in the striatum, which subsequently resulted in lower preference for high-fat diets and reduced body weight (47). Our study revealed that exercise has the potential to restore striatal insulin sensitivity in humans. Based on these findings, central dopamine signaling is a potential target for interventions in the fight against obesity and T2D.

Enhanced neural plasticity in the hippocampus is among the first exercise-mediated improvements in the brain (56–58). In line with these findings, we observed heightened insulin action in the right hippocampus in response to exercise. After the exercise program, FC between the right hippocampus and prefrontal areas of the DMN responded to insulin, comparable with what we previously detected in lean healthy adults (15). This appears to result in clinically relevant improvements, as the restored insulin-dependent FC was linked to better cognitive function after the exercise intervention. This is well in line with a recent metaanalysis that underlined aerobic exercise’s effects in improving cognitive flexibility (59).

Beyond the brain, our results demonstrate a significant correlation between increased mitochondrial respiration in the skeletal muscle and restored brain insulin sensitivity in response to exercise training. Persons with the greatest improvement in skeletal muscle mitochondrial respiration show the most prominent increase in striatal insulin action as measured by CBF. Mediation analyses even suggest that exercise effects on energy metabolism in the skeletal muscle may be a starting point of a complex process that depends on the brain and results in improved whole-body metabolism. Hereby, the improved striatal insulin responsiveness served as a significant mediator between skeletal muscle mitochondrial function and the changes in hunger and body fat distribution. Increased mitochondrial respiration in skeletal muscle is a repeatedly reported effect of exercise training in humans (60). Recent data from rodents show improvement of mitochondrial function after exercise in the brain. This was accompanied by enhanced brain insulin action (61). It can be speculated that improved mitochondrial respiration in skeletal muscle is reflective of exercise effects on brain mitochondrial function, with enhanced brain ATP production contributing to restored brain insulin sensitivity. Furthermore, the trained skeletal muscle releases peptides and other exercise factors, which may communicate exercise effects to the brain (62). Further investigations are necessary to clarify the mechanisms at each individual step from the exercising skeletal muscle to brain insulin sensitivity and the consequences for peripheral metabolism and behavior. Moreover, based on recent findings, there are individuals with marked improvements in whole-body insulin sensitivity after exercise and those with little or no improvement (48, 49). Our work gives ground to investigate the contribution of the brain to the responsiveness to exercise in future studies. How long the beneficial effects of exercise on brain insulin action are retained and if the brain converts back to an insulin-resistant state after resuming a sedentary lifestyle is also an important open question for future research. Furthermore, we identified selective improvements of brain insulin responsiveness in the mesolimbic system without identifying changes in hypothalamic insulin action. This may be due to the type of intervention, as exercise was previously reported to particularly modulate dopaminergic (47, 55) and hippocampal function (56). Whether weight loss or hypocaloric diet improves brain insulin responsivity in a comparable fashion is currently not known. A more detailed understanding of this complex process can guide the design of comprehensive intervention programs with optimal benefits for brain health and metabolism.

There are limitations to our study, such as the lack of a formal randomized control group. Hence, we cannot rule out practice and repetition effects. Nevertheless, in this exploratory research, we were able to analyze and compare brain insulin action of participants who were overweight or obese from previous studies without an exercise intervention. Moreover, sex-specific findings have been identified on brain insulin responsivity (20, 21, 63), which could not be investigated in the current study based on the limited sample size. The correlative nature of the findings prohibits us to clarify mechanisms of actions. One such mechanism can also involve alterations in central insulin transport and distribution, as was demonstrated in mice after a single bout of exercise (58). However, the 7 days between the last bout of exercise and the fMRI measurements makes acute effects of exercise on insulin transport into the brain unlikely. In our study, we used nasal insulin administration to probe brain insulin action once before and once after the 8-week training intervention. There is a well-characterized spillover of small amounts of nasal insulin into the circulation that is cleared after approximately 30 minutes after nasal spray (14). This tiny rise in circulating insulin is not sufficient to cause hypoglycemia or suppress C-peptide levels (14). Even though it is unlikely, we cannot fully rule out that this might have impacted our results — e.g., by temporarily suppressing lipolysis (29, 64). Even though not central for the findings reported here, it is a limitation that peripheral insulin sensitivity was estimated from OGTTs but not determined by hyperinsulinemic euglycemic glucose clamp.

In conclusion, an 8-week aerobic exercise training intervention can improve brain insulin sensitivity that was associated with beneficial exercise-induced effects in metabolism and behavior. This study suggests that brain IR may not be a fixed trait but a viable therapeutic target to counteract the repercussions of obesity upon cognition and metabolism. Restored insulin action in the brain elicits benefits for the entire body that translate to clinically relevant outcomes. Further controlled intervention studies are needed to validate whether improving insulin sensitivity of the brain in people at high risk to develop T2D truly has beneficial effects on metabolism and cognition and to elucidate the underlying mechanisms.

## Methods

### Study design and participants.

Twenty-six healthy, sedentary individuals at elevated risk for T2D were recruited to participate in an 8-week supervised endurance training intervention program. Details of this intervention study and the preregistered primary endpoint have been recently published (48). Inclusion and exclusion criteria are described in Supplemental Methods. Briefly, 3 times a week, participants performed a 1-hour supervised endurance training session, which included a combination of cycling and walking training at 80% VO_2_peak. Heart rate corresponding to 80% VO_2_peak was used to standardize exercise intensity. Cardiopulmonary exercise testing with breath gas analysis and assessment of capillary lactate concentrations was used to analyze physical fitness as VO_2_peak and the individual anaerobic threshold (IAT), respectively. Peripheral insulin sensitivity was estimated from 5-point 75 g OGTT, and body fat distribution was quantified using whole-body MRI measurements (Figure 1). Skeletal muscle biopsies (vastus lateralis) were taken 60 minutes after an acute exercise bout before and after 8-week acute exercise (Supplemental Figure 3). High-resolution respirometry (Oroboros Oxygraph 2k; Oroboros Instruments GmbH) was used to analyze mitochondrial respiration in single, permeabilized myofibers. Maximal coupled respiration was measured after addition of malate (1.28 mM), octanoylcarnitine (0.5 mM), ADP (2.5 mM), sodium pyruvate (5 mM), and succinate (2.5 mM). Maximal uncoupled respiration was measured after additional cytochrome c (10 μM) and titration with FCCP (125 nM steps) (48).

Twenty-one participants were eligible for fMRI measurements (14 women, 7 men; age 21–59 years; BMI 27.5–45.5 kg/m^2^) (Table 1). Brain insulin action was assessed by fMRI before and 1 week after the 8-week exercise intervention. OGTT and brain insulin action assessments were performed 2 days apart.

### Whole-brain fMRI measurement.

Brain insulin action was quantified before and after the exercise intervention by application of intranasal insulin in combination with fMRI recordings (Figure 1). By the intranasal method, insulin can bypass the body periphery and directly enter the CNS (65); this facilitates the differentiation between peripheral and central insulin effects. Measurements were performed after an overnight fast of at least 10 hours and started at 7 a.m. with fMRI measurement under fasting conditions (fMRI-1). After the basal fMRI measurement, 160 U of human insulin spray was administered intranasally by spraying 2 puffs per nostril (each containing 10 U of insulin) every minute over 4 minutes (Insulin Actrapid; Novo Nordisk). Thirty minutes later, a second fMRI measurement was performed (fMRI-2). At this time point, serum insulin concentrations are comparable to baseline levels prior to spray (14), following a transient absorption of around 0.1 U of the intranasally administered insulin into the bloodstream (66).

Perceived feeling of hunger (VAS from 0 [not hungry at all] to 10 [very hungry]) was rated before insulin spray application and 60 minutes thereafter. After the second fMRI measurement, cognitive function was evaluated by the trail making test (TMT). This measure of cognitive flexibility has been shown to improve in response to aerobic exercise (59).

### Data acquisition.

Scanning was conducted at a 3T whole-body Siemens scanner (Magnetom Prisma) with a 20-channel coil. Two different types of functional data sets were recorded at each visit before and after nasal spray application. In addition, high-resolution T1-weighted anatomical images were obtained.

To acquire CBF maps, pulsed arterial spin labeling (PASL) images were obtained with a PICORE-Q2TIPS sequence. To assess resting-state FC, whole-brain blood-oxygen-level-dependent data were collected by using multiband accelerated echoplanar imaging sequences, developed at Center for Magnetic Resonance Research (CMRR; Minneapolis, Minnesota, USA). For detailed sequence parameters, see Supplemental Methods.

### ASL Image processing.

Image preprocessing was performed by using the ASLtbx with SPM12 (Wellcome Trust Centre for Neuroimaging). Functional images were motion corrected, coregistered to the individual anatomical image, and smoothed (full width at half maximum, 6 mm). Perfusion images were generated by calculating the control-tag differences by using surround subtraction. For accurate CBF quantification (mL/100 g/min), we used unique M0 value extracted from a ROI in the cerebro spinal fluid (CSF). We used the general kinetic model for absolute perfusion quantification. Recent reliability studies using ASL (67) and our current measurements showed high reproducibility and reliability. For more details, see Supplemental Methods.

### Resting-state fMRI data processing.

We used the Data Processing Assistant for Resting-State fMRI (68) to analyze the resting state fMRI data, which is based on SPM12 and Resting-State fMRI Data Analysis Toolkit. The whole-brain functional images were normalized to voxel size: 2 × 2 × 2 mm^3^ and then smoothed (full width at half maximum, 6 mm). Nuisance regression was performed using white matter, CSF, and the 6 head-motion parameters as covariates.

FC maps were obtained using a seed-based voxel-wise correlation approach by computing FC between a seed region and each voxel within the brain. We used the core regions of the DMN according to our recent publication investigating central insulin effects on FC (15). The seeds included the following coordinates with a 5 mm sphere (seed 1: *x*, –6; *y*, 52; *z*, -2; seed 2: *x*, 0; *y*, 52; *z*, 26; seed 3: posterior cingulate/precuneus *x*, –8; y, –56; *z*, 26). The FC maps were transformed to *Z* values using Fisher’s transformation.

### Whole-body MRI for quantification of adipose tissue compartments.

T1-weighted fast spin-echo images with a slice thickness of 1 cm and an interslice gap of 1 cm were acquired from the entire body on a 3T MAGNETOM Vida MRI (Siemens Healthineers) in the early morning after overnight fasting as described in ref. 69.

### Data availability.

Due to the potential to compromise research participant privacy/consent, the data will only be made available to interested researchers upon reasonable request.

### Statistics.

Paired *t* tests (2-tailed) were carried out to evaluate differences from before to after the exercise intervention on brain insulin responsiveness, as well as behavioral and metabolic data. Additionally, exploratory correlation and mediation analyses were performed between exercise-induced changes in brain insulin responsiveness, behavioral and peripheral/metabolic data.

Regarding metabolic data, paired 2-tailed *t* tests were carried out on anthropometric measures, whole-body MRI, and mitochondrial respiration (*P* < 0.05 considered significant; Table 1 and Supplemental Table 1) (SPSS version 27).

Regarding brain data, the primary analysis was performed to assess brain insulin responsiveness using CBF and FC changes before (pre) and after the 8-week exercise intervention (Supplemental Figure 1). To this end, CBF and FC whole-brain maps of each participant were corrected for baseline measurements to determine the effect of central insulin action before (ΔCBF_pre_ = CBF_fMRI-2_ – CBF_fMRI-1_) (ΔFC_pre_ = FC_fMRI-2_ – FC_fMRI-1_) and after the 8-week intervention (ΔCBF_post-8-week_ = CBF_fMRI-2_ – CBF_fMRI-1_) (ΔFC_post-8-week_= FC_fMRI-2_ – FC_fMRI-1_). Whole-brain analyses were performed using a voxel-wise approach in SPM12. Paired *t* tests were carried out to investigate the difference of ΔCBF_pre_ versus ΔCBF_post-8-week_ and ΔFC_pre_ versus ΔFC_post-8-week_. Moreover, CBF and FC maps of each measurement time point of each participant (CBF/FC_fMRI-1_ and CBF/FC_fMRI2_ nasal spray before and after the 8-week exercise intervention) were entered into a flexible factorial design to determine the effect of exercise on CBF and FC independently of intranasal insulin. A statistical threshold of *P* < 0.001 whole-brain uncorrected and a *P*_FWE_ < 0.05 corrected for multiple comparisons was applied. SVC was additionally applied for regions previously reported as insulin sensitive (mask based on ref. 2) (SVC *P*_FWE_ < 0.05*)*.

Regarding behavioral data, perceived feeling of hunger ratings were corrected for baseline measurements to determine the central insulin effects on hunger before (ΔVAS_pre_ = VAS_fMRI-2_ – VAS_fMRI-1_) and after the 8-week intervention (ΔVAS_post-8-week_= VAS_fMRI-2_ – VAS_fMRI-1_). This differential measure was used for correlation analyses. Additionally, a paired *t* test was carried out to investigate the difference of pre– versus post–8-week hunger ratings.

TMT A and B scores were assessed once before and after the intervention. A paired *t* test was carried out to investigate the difference from before to after the intervention (SPSS version 27).

Regarding correlation analyses, we extracted CBF and FC values of significant clusters to perform exploratory correlation analyses with an exercise-induced change in behavioral and peripheral/metabolic data.

Exploratory mediation analyses were performed using PROCESS version 4.0 procedure in SPSS (by Andrew F. Hayes). The significance of the mediation analysis (i.e., indirect effect *ab*) was estimated based on a bias-corrected bootstrap CI (95% CI, 5,000 bootstrap samples). For all correlation analyses, *P* < 0.05 was considered significant (SPSS version 27).

### Study approval.

All participants gave written informed consent prior to participating in the study, and the study protocol was approved by the local ethics committee of the University of Tübingen.

## Author contributions

SK analyzed most of the data, interpreted the data, and wrote manuscript with the contribution from all coauthors. TG analyzed mitochondrial respiration data. RV analyzed CBF data and provided scientific guidance. LF collected metabolic data. LW performed fMRI experiments and collected behavior data. PS conduced exercise training. M Hoene contributed to data interpretation and discussion. CH analyzed mitochondrial respiration data. JM performed whole-body MRI. AN designed the study and contributed to discussion. HP, AF, ALB, and AP provided scientific guidance and experimental design, and they contributed to discussion. HUH secured funding and designed study. AM designed the study and analyzed metabolic data. CW and M Heni designed the study, supervised the project, interpreted the data, wrote the manuscript, and are the guarantors of this work.

## Supplementary Material

Supplemental data

ICMJE disclosure forms

## Figures and Tables

**Table 1:**
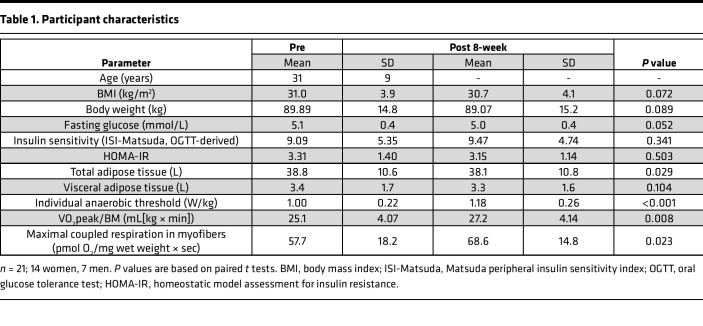
Participant characteristics

## Version 1 09/22/2022

Electronic publication
