# Supplementary material for: Exercise restores brain insulin sensitivity in sedentary adults who are overweight and obese
Source: JCI Insight. 2022 Sep 22;7(18):e161498. doi: 10.1172/jci.insight.161498 (PMC9675563; doi:10.1172/jci.insight.161498)

Supplementary material

Supplementary methods

Participants

Healthy, sedentary (<120 minutes of physical activity per week) individuals at high risk for T2D with at least one of the following risk factors (BMI >27 kg/m2, family history (first degree) of T2D, former gestational diabetes) were recruited. Twenty-six subjects met the study criteria and completed the study. One participant was excluded from data analysis due to newly diagnosed autoimmune thyroiditis. Severe diseases were excluded by anamnesis including medication, monitoring routine laboratory parameters, electrocardiogram, and physical examination. 21 participants (14 women) were edible for fMRI measurements.

MRI Data acquisition

To acquire CBF maps, arterial spin labeling (PASL) images were obtained with a PICORE-Q2TIPS (proximal inversion with control for off-resonance effects—quantitative imaging of perfusion by using a single subtraction) sequence by using a frequency offset corrected inversion pulse and echo planar imaging readout for acquisition. A total of 16 axial slices with a slice thickness of 4.5 mm (0.90-mm gap) were acquired in ascending order. Each measurement consisted of 79 alternating tag and control images with the following imaging parameters: inversion time (TI), TI1 = 700 ms, TI2 = 1800 ms, repetition time (TR) = 3000 ms, echo time = 13 ms, inplane resolution = 3x3 mm^2, field of view = 192 mm, matrix size 64x 64 and flip angle = 90°.To assess resting-state functional connectivity, whole-brain BOLD data were collected by using multi-band accelerated echo-planar imaging sequences, developed at Center for Magnetic Resonance Research (CMRR) Minnesota, USA. The following sequence parameters were used: TR = 1.5s, TE = 34ms, FOV = 192 mm^2, matrix 96 x 96, partial Fourier = 6/8, bandwidth = 2264 Hz/pixel, echo spacing = 0.55 ms, flip angle 70°, voxel size 2 x 2 x 2 mm^3, slice thickness 2 mm, images were acquired in interleaved order with a multiband acceleration factor of 3.

ASL Image processing

Image preprocessing was performed by using the ASLtbx (1) with SPM12 (Wellcome Trust Centre for Neuroimaging). Functional images were motion corrected, coregistered to the individual anatomical image and smoothed (full width at half maximum: 6mm). Perfusion images were generated by calculating the control-tag differences by using surround

subtraction. For accurate CBF quantification (ml x $100g^{-1}$ x min^{-1}), we used a unique M0 value extracted from a ROI in the cerebro spinal fluid. For absolute perfusion quantification the general kinetic model was applied. Possible outliers were cleaned using a slice-wise procedure based on priors (2). The high resolution T1-weighted image was normalized in Montreal Neurological Institute space (1x1x1mm) using SPM12's unified segmentation normalization, and the resulting parameter file was used with the individual co-registered CBF maps in normalized space (3x3x3mm). A brain mask was used to exclude extracranial voxels in the normalized CBF images.

Reproducibility and reliability of global and putamen cerebral blood flow was characterized in 110 healthy individuals (49.1% women, 46.4% overweight and obese, age range 21 – 74 years, BMI range 18 – 49 kg/m^2) with available cerebral blood flow measurements, as acquired and analyzed in the current study. Participants were enrolled in studies from 2014 to 2018 with two measurement time points separated by 2 to 6 weeks without a lifestyle or pharmaceutical intervention in between. Reproducibility was characterized using the within-subject coefficient of variation (CV). Reliability was measured using a two way mixed model intraclass correlation coefficient (ICC range: 0-1, values >0.75 are classified as excellent reliability(3)). There was no significant difference between measurement days for global and putamen CBF ($p>0.05$, paired t-test). Reproducibility and reliability were very high, indicated by low CV (CV_{global}= 0.06±0.05, $CV_{putamen}$= 0.09±0.06) and high ICC values (ICC_{global}= 0.904, $ICC_{putamen}$= 0.875), which is comparable with a recent reliability study highlighting the potential of ASL for longitudinal studies (4, 5).

Supplementary table 1.

**Mitochondrial respiration of skeletal muscle fibers**					
	**Pre**		**Post-8-wk**		p-value
	mean	SD	mean	SD	
Respiration on octanoylcarnitine and pyruvate[1]	43.30	15.69	53.51	11.09	0.008
Maximal coupled respiration[2]	57.68	18.21	68.63	14.83	0.023
Maximal uncoupled respiration[3]	77.53	24.33	92.90	22.56	0.022

P-values are based on paired t-tests. [1]measured after addition of malate + octanoylcarnitine + ADP + pyruvate; [2]measured after addition of malate + octanoylcarnitine + ADP + pyruvate+ succinate; [3]after titration with Carbonyl cyanide-p-trifluoromethoxyphenylhydrazone (FCCP), see Methods section for details; SD, standard deviation.

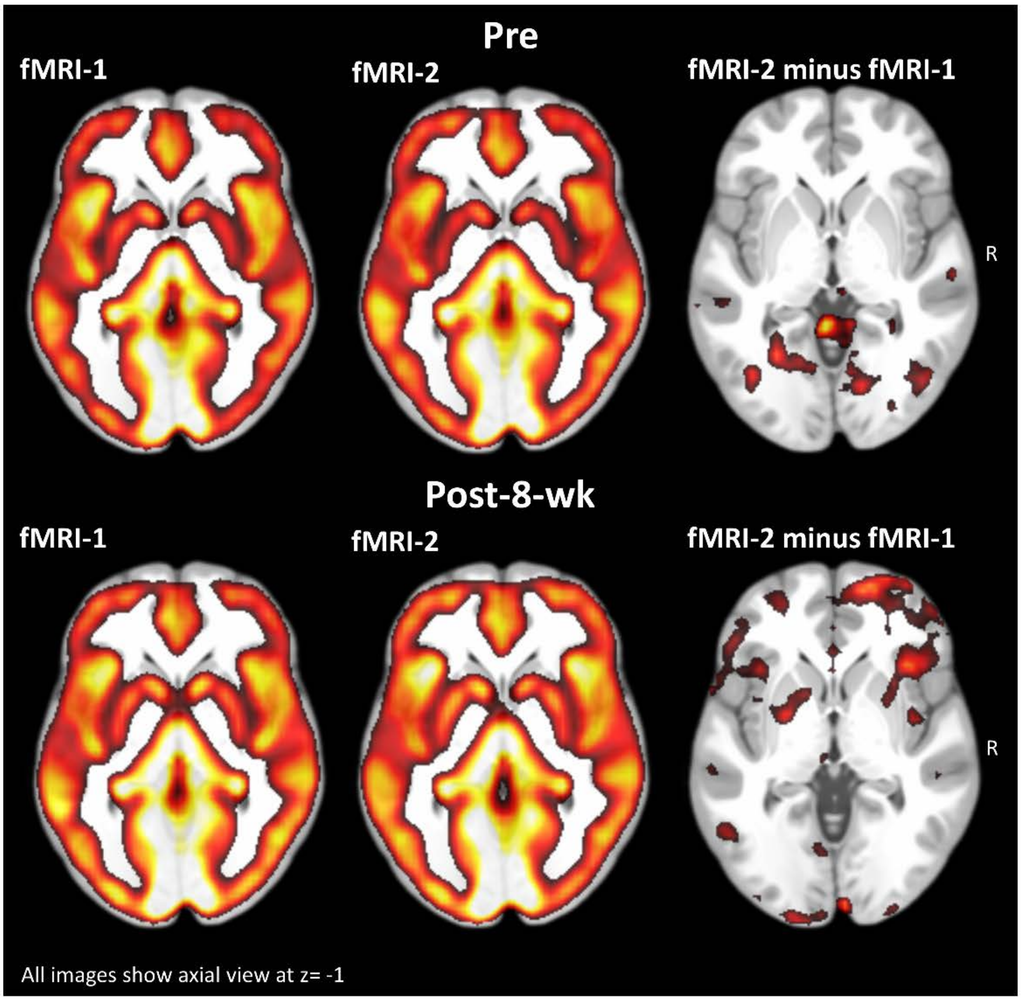

Supplementary Figure 1. Group averaged whole-brain resting-state cerebral blood flow (ml/100g/min) on a T1-weighted template brain in the axial view (z=-1) shown for descriptive purposes only. Each session displayed was the mean CBF of all participants for fMRI-1 (before nasal spray) and fMRI-2 (30 min after insulin nasal spray) on the two measurement days before (pre) and after the 8 week exercise intervention. The fMRI-1 and fMRI-2 image show a CBF range of 30 to 60 ml/100g/min. The fMRI2 minus fMRI-1 image shows the CBF difference of 3 to 10 ml/100g/min.

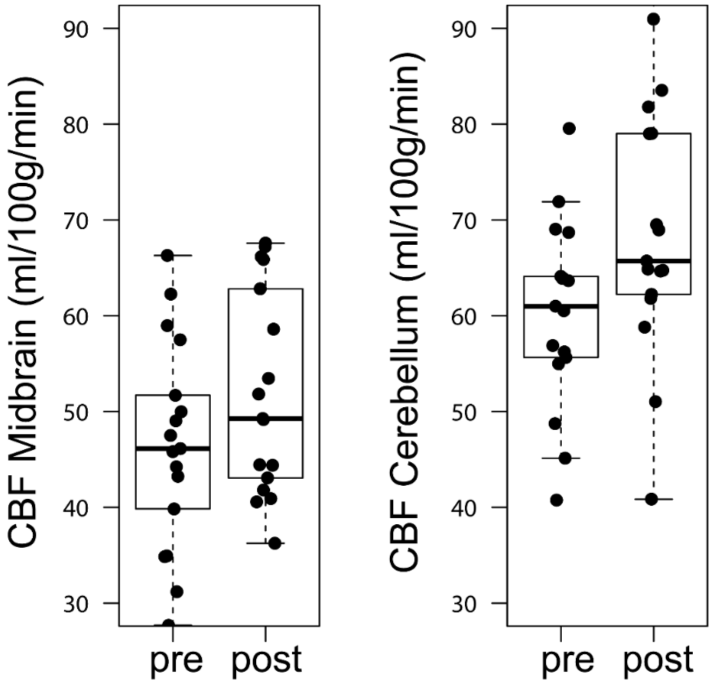

Supplementary figure 2: Increased cerebral blood flow after exercise intervention. The box plots show cerebral blood flow (CBF) in the midbrain (left side) and cerebellum (right side) pre and post 8-wks exercise intervention ($p<0.05$ FWE corrected). In the box plot, the box indicates the first and third quartile (25th and 75th percentile), the line in the box marks the median, whiskers above and below indicate 1.5* interquartile range.

Supplementary table 2. Effect of exercise on cerebral blood flow before and after insulin nasal spray

Brain region	MNI coordinate	T value	P Value
**Main effect of exercise CBF $_{pre}$ < CBF $_{post-8-wk}$**			
Midbrain	0 -22 -16	4.76	<0.05*
Left cerebellum	-3 -49 -16	4.43	<0.05*
**Paired T-test of Δ CBF $_{pre}$ < Δ CBF $_{post-8-wk}$**			
Right putamen	30 8 -1	4.05	<0.05§
Left putamen	-24 -1 -1	3.26	n.s.

*FWE corrected on whole-brain level; § small volume correction; n.s.: not significant

Supplementary table 3. Regional cerebral blood flow in the putamen (ml/100g/min)

	**fMRI1 (baseline)**		**fMRI2 (30 min after insulin)**		**fMRI2 minus fMRI1**	
	mean	SD	mean	SD	mean	SD
**Exercise intervention study (n=18)**						
Pre	33.76	9.99	31.88	6.75	-1.43	7.15
Post-8-weeks	35.65	10.88	38.76	7.79	4.52	7.64
**Comparison group (oral placebo intake; n=20; overweight and obese)(6)**						
Pre	37.36	6.42	36.14	8.22	-1.22	4.68
Post-8-weeks	34.69	9.17	34.19	9.78	-0.45	5.08
**Cross sectional comparison group (n=34; normal weight, overweight and obese)(7)**						
Normal-weight	41.58	11.06	46.16	9.48	3.36	9.04
Overweight/ obese	37.77	10.48	38.17	12.97	-1.99	0.68

Supplementary table 4. Hunger ratings and cognitive performance

	**Pre**		**Post-8-wk**		T value Df=20	p-value
	mean	SD	mean	SD		
**VAS Hunger fMRI-1**	4.99	2.53	4.31	2.40	1.2	0.23
**VAS Hunger fMRI-2**	5.86	2.62	5.46	2.83	0.78	0.44
**ΔVAS (fMRI-2 minus fMRI-1)**	0.87	2.21	1.14	2.51	-0.47	0.642
**TMT-A [sec]**	25.02	7.09	21.78	5.77	1.7	0.09
**TMT-B [sec]**	49.37	11.964	40.65	6.98	4.81	0.0001

Abbreviations: fMRI-1: before nasal spray, fMRI-2: after nasal spray; VAS: visual analog scale; TMT: trial-making test.

Supplementary table 5. Results of mediation models

Independent variable (IV)	Dependent variable (DV)	Mediator	Model statistics			
			Coeff (SE)	Std. Coeff (Boot SE)	CI 95%	
					LLCI	ULCI
**Models using fold change in mitochondrial respiration as predictor (IV)**						
FC maximal coupled respiration	FC VAT	ΔΔ CBF putamen				
Path *a*			11.1 (2.9)	.534	4.86	17.4
Path *b*			.011 (.004)	-.568	-.021	-.001
Total effect (c)			-.155 (.086)	-.384	-.340	.030
Direct effect (c’)			-.032 (.084)		-.215	.150
**Indirect effect (*ab*)**			**-.122 (.078)**	**-.304**	**-.719**	**-.032**
FC maximal coupled respiration	ΔΔ Hunger	ΔΔ CBF putamen				
Path *a*			33.7 (10.6)	.535	11.05	56.5
Path *b*			-.253 (0.05)	-.820	-.362	-.144
Total effect (c)			-1.9 (3.2)	-.09	-8.9	5.12
Direct effect (c’)			6.6 (3.8)		-1.8	15.0
**Indirect effect (*ab*)**			**-8.5 (4.2)**	**-.439 (.209)**	**-.871**	**-.05**
**Models using fold change in visceral adipose tissue as a predictor (IV)**						
FC VAT	ΔΔ Hunger	ΔΔ CBF putamen				
Path *a*			-32.6 (7.87)	-.602	-49.4	-15.8
Path *b*			-.241 (0.05)	-.821	-.357	-.126
Total effect (c)			2.08 (4.5)	.131	-7.59	11.7
Direct effect (c’)			-5.7 (3.6)		-13.57	2.0
**Indirect effect (*ab*)**			**7.8 (2.7)**	**.495 (.163)**	**.150**	**.813**

Models reporting a significant indirect effect between the IV and DV mediated by the change in insulin action in the putamen (M). Abbreviations: CBF, cerebral blood flow; CI, confidence interval; FC, fold change; SE, standard error; VAT, visceral adipose tissue; ΔΔ means (Δpre = fMRI-2 – fMRI-1) minus (Δpost-8-wk = fMRI-2 – fMRI-1).

Supplementary results

To account for the lack of a control group in the current study, we extracted the insulin response of the right putamen from the oral placebo control group (n=20) of a previously published study with the same CBF acquisition method in response to intranasal insulin but without an exercise intervention (6, 7) (see supplementary table 3).

Exploratory between group analyses showed a similar insulin response in the right putamen for the baseline visit (T(35)= 1.09, p=0.280) and a statistical significant difference for the follow up visit (post-8-weeks) (T(35)= -2.3, p= 0.025) between participants of the exercise intervention and the comparison group.

Additionally, we extracted the insulin response of the right putamen in 34 age-matched participants of normal, overweight and obesity from a cross-sectional study (7). Prior to the exercise intervention (at baseline), participants of the current study showed a comparable central insulin response in the putamen as the participants with overweight and obesity (T(33)= 0.38, P=0.706) but a significant difference compared to the normal weight group of the previous study cohort (T(33)= 2.45, p=0.02). After the exercise intervention, participants of the current study showed a similar increase to central insulin as persons of normal-weight (T(33)= -0.4, p=0.692) (figure 3D). Hence, 8 weeks of endurance exercise led to a change in the insulin response in putamen in individuals with overweight to obesity, which is comparable to the insulin response in this brain region of normal weight persons.

References

1. Wang Z, Aguirre GK, Rao H, Wang J, Fernandez-Seara MA, Childress AR, et al. Empirical optimization of ASL data analysis using an ASL data processing toolbox: ASLtbx. *Magn Reson Imaging.* 2008;26(2):261-9.
2. Li Y, Dolui S, Xie DF, Wang Z, and Alzheimer's Disease Neuroimaging I. Priors-guided slice-wise adaptive outlier cleaning for arterial spin labeling perfusion MRI. *J Neurosci Methods.* 2018;307:248-53.
3. Shrout PE, and Fleiss JL. Intraclass correlations: uses in assessing rater reliability. *Psychol Bull.* 1979;86(2):420-8.
4. Ssali T, Anazodo UC, Bureau Y, MacIntosh BJ, Gunther M, and St Lawrence K. Mapping Long-Term Functional Changes in Cerebral Blood Flow by Arterial Spin Labeling. *PLoS One.* 2016;11(10):e0164112.
5. Murphy K, Harris AD, Diukova A, Evans CJ, Lythgoe DJ, Zelaya F, et al. Pulsed arterial spin labeling perfusion imaging at 3 T: estimating the number of subjects required in common designs of clinical trials. *Magn Reson Imaging.* 2011;29(10):1382-9.
6. Kullmann S, Hummel J, Wagner R, Dannecker C, Vosseler A, Fritsche L, et al. Empagliflozin Improves Insulin Sensitivity of the Hypothalamus in Humans With Prediabetes: A Randomized, Double-Blind, Placebo-Controlled, Phase 2 Trial. *Diabetes Care.* 2022;45(2):398-406.
7. Kullmann S, Heni M, Veit R, Scheffler K, Machann J, Haring HU, et al. Selective insulin resistance in homeostatic and cognitive control brain areas in overweight and obese adults. *Diabetes Care.* 2015;38(6):1044-50.

Supplementary Figure 3

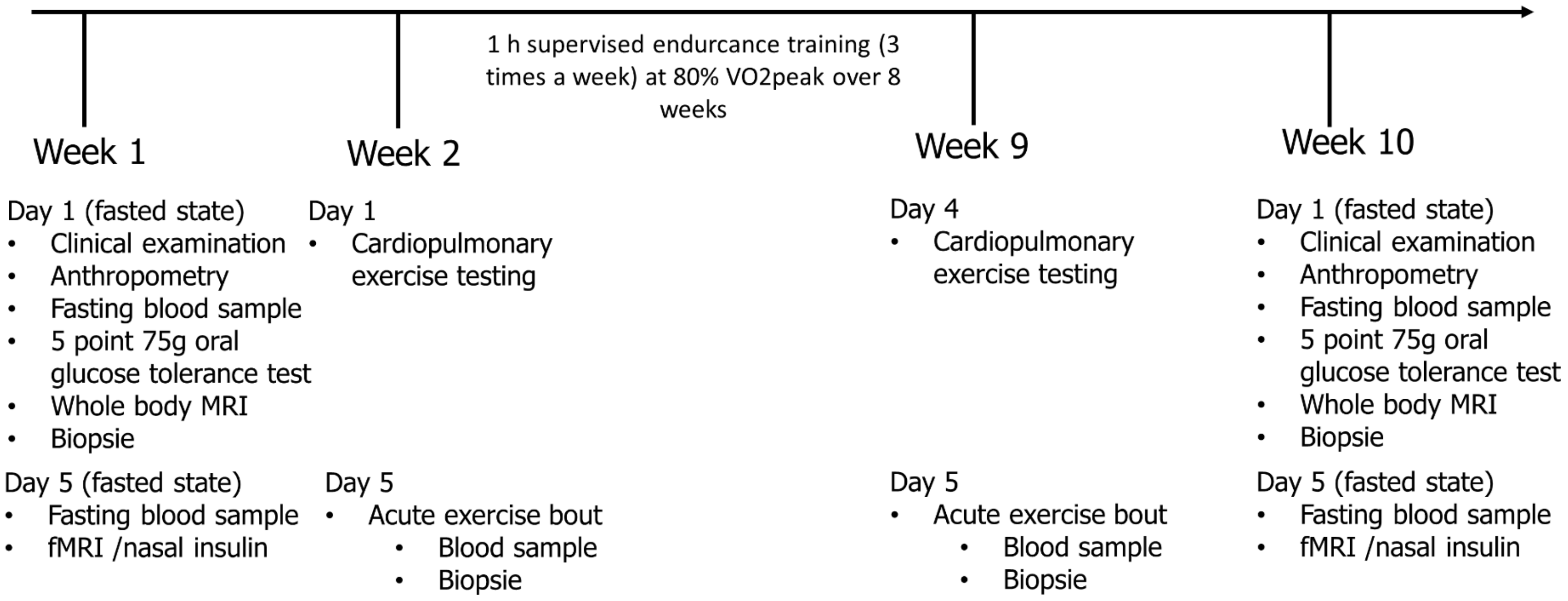

Supplement: Supplemental data [file jciinsight-7-161498-s033.pdf]
